# Capture Hi-C reveals the influence on dynamic three-dimensional chromosome organization perturbed by genetic variation or vanillin stress in *Saccharomyces cerevisiae*

**DOI:** 10.3389/fmicb.2022.1012377

**Published:** 2022-11-17

**Authors:** Xinning Wang, Bolun Yang, Weiquan Zhao, Wenyan Cao, Yu Shen, Zailu Li, Xiaoming Bao

**Affiliations:** ^1^State Key Laboratory of Biobased Material and Green Papermaking, School of Bioengineering, Qilu University of Technology (Shandong Academy of Sciences), Jinan, China; ^2^State Key Laboratory of Microbial Technology, Institute of Microbial Technology, Shandong University, Qingdao, China; ^3^Shandong University Library, Jinan, China

**Keywords:** vanillin, Hi-C, topologically associating domain, *Saccharomyces cerevisiae*, stress resistance, mutation

## Abstract

Studying the mechanisms of resistance to vanillin in microorganisms, which is derived from lignin and blocks a major pathway of DNA double-strand break repair in yeast, will benefit the design of robust cell factories that produce biofuels and chemicals using lignocellulosic materials. A high vanillin-tolerant *Saccharomyces cerevisiae* strain EMV-8 carrying site mutations compared to its parent strain NAN-27 was selected for the analyses. The dynamics of the chromatin structure of eukaryotic cells play a critical role in transcription and the regulation of gene expression and thus the phenotype. Consequently, Hi-C and transcriptome analyses were conducted in EMV-8 and NAN-27 in the log phase with or without vanillin stress to determine the effects of mutations and vanillin disturbance on the dynamics of three-dimensional chromosome organization and the influence of the organization on the transcriptome. The outcomes indicated that the chromosome interaction pattern disturbed by vanillin stress or genetic mutations in the log phase was similar to that in mouse cells. The short chromosomes contact the short chromosomes, and the long chromosomes contact the long chromosomes. In response to vanillin stress, the boundaries of the topologically associating domain (TAD) in the vanillin-tolerant strain EMV-8 were more stable than those in its parent strain NAN-27. The motifs of *SFL1*, *STB3*, and *NHP6A/B* were enriched at TAD boundaries in both EMV-8 and NAN-27 with or without vanillin, indicating that these four genes were probably related to TAD formation. The Indel mutation of *YRR1*, whose absence was confirmed to benefit vanillin tolerance in EMV-8, caused two new interaction sites that contained three genes, *WTM2*, *PUP1*, and *ALE1*, whose overexpression did not affect vanillin resistance in yeast. Overall, our results revealed that in the log phase, genetic mutations and vanillin disturbance have a negligible effect on three-dimensional chromosome organization, and the reformation or disappearance of TAD boundaries did not show an association with gene expression, which provides an example for studying yeast chromatin structure during stress tolerance using Hi-C technology.

## Introduction

As a plentiful and renewable biomass resource, lignocellulosic materials are widely studied for the production of biofuels and other valuable chemicals. Pretreatment of lignocellulosic materials to destroy the complex structure of lignocellulose and release monosaccharides is crucial for the efficient utilization of lignocellulose (Maurya et al., 2015; Ramos and Duque, 2019). However, this process can generate a series of inhibitors, such as organic acids, furans, and phenolics, which inhibit the growth and metabolism of microorganisms (Saini et al., 2015; Sindhu et al., 2016).

Vanillin, a typical guaiacyl phenol generated by lignin degradation during pretreatment, is considered an important inhibitor of lignocellulosic hydrolysates because it inhibits the viability of many microorganisms at very low concentrations (Klinke et al., 2004). The concentrations of vanillin range from 1 to 26 mM according to the types of biomass materials and the method of pretreatment (Almeida et al., 2007; Heer and Sauer, 2008). In addition, as a natural product with a unique scent, vanillin is the second most demanded flavoring agent after saffron and is used in food products, perfumery, beverages, and the pharmaceutical industry due to its biological activities, such as its scent and its antioxidant, antitumorigenic, tranquilizer, and antidepressant activities (Walton et al., 2000; Tai et al., 2011; Banerjee and Chattopadhyay, 2019; Bettio et al., 2021; Zhang et al., 2021). The toxicity of vanillin to microbial cell factories such as *Escherichia coli* and *S. cerevisiae* restricts its biosynthesis. Heterologous biosynthesis of vanillin using ferulic acid or glucose as a substrate has become increasingly attractive, as natural vanillin from vanilla pod extracts cannot meet the increasing demand (Hansen et al., 2009; Brochado et al., 2010; Kunjapur et al., 2016; Liang et al., 2020). Thus, exploring the cellular protective mechanisms related to vanillin resistance is important for the efficient utilization of lignocellulosic materials or the efficient production of vanillin.

Vanillin was reported to disrupt the integrity of biological membranes, thereby decreasing cell growth (Liang et al., 2020), repressing translation by affecting the function of the large ribosomal subunit (Iwaki et al., 2013), and inducing oxidative stress and mitochondrial fragmentation (Nguyen et al., 2014). Moreover, vanillin was reported to block DNA end-joining by directly inhibiting the activity of DNA-PK, a crucial enzyme of non-homologous DNA end-joining (NHEJ), which is a major pathway of double-strand break (DSB) repair in human cells (Durant and Karran, 2003). Endo et al. (2008) found that deletion mutations associated with chromosome remodeling and vesicle transport caused vanillin sensitivity by screening 76 *Saccharomyces cerevisiae* deletion mutants that were sensitive to vanillin and performing cluster analysis on these deletion genes. They conjectured that vanillin might cause DNA breakdown and block the subsequent DNA repair process, leading to serious DNA damage (Endo et al., 2008). Based on the above findings, we speculated that vanillin might influence the 3D organization of chromosomes.

Most of the DNA in eukaryotic cells is stored in the nucleus in the form of chromosomes, long thread-like structures. The dynamics of higher-order chromatin structure influence the transcription, replication, and repair of DNA and the regulation of gene expression (Chen and Li, 2010). The yeast *Saccharomyces cerevisiae*, a well-studied model system for understanding fundamental cellular processes of corresponding higher eukaryotic organisms, is widely used in traditional ethanol production cell factories because of its easy genetic manipulation and robustness to exposure to ethanol and low pH (Galao et al., 2007; Gibson et al., 2007; Liu et al., 2009; Hong and Nielsen, 2012). It is also a good model for the three-dimensional organization of eukaryotic genomes (Kim et al., 2017). In our previous study, EMV-8, a prominently higher vanillin-tolerant *S. cerevisiae* strain than its parent strain NAN-27, which is an ethanol-producing strain used by Henan Tianguan Group Co., Ltd. (China), was obtained through ethyl methanesulfonate (EMS) mutation and adaptive evolution in lignocellulosic hydrolysates (Shen et al., 2014). To obtain a global view of the dynamics of three-dimensional chromosome organization affected by vanillin stress and mutations of EMV-8, as well as the influence of 3D chromosome organization on the transcriptome, we conducted an evaluation using a high-throughput chromosome conformation capture technology, Hi-C, and transcriptomic analysis between NAN-27 and EMV-8 with or without vanillin stress.

## Materials and methods

### Strains, plasmids, and culture conditions

The Hi-C assay captured the chromosome conformations of the *S. cerevisiae* industry strain NAN-27 (Zhang et al., 2010) and its derivative strain EMV-8, a vanillin-tolerant strain (Shen et al., 2014). The genes for overexpression were amplified from EMV-8 and expressed in laboratory strain BY4741 (*MATa, his3*Δ*1 leu2*Δ *met5*Δ *ura3*Δ, EUROSCARF, Germany). pJFE3 (Shen et al., 2012), a 2 μ plasmid with a *TEF1* promoter, a *PGK1* terminator, and *URA3* as the selection marker, was used as a vector for gene overexpression.

Yeast extract peptone dextrose (YEPD) medium (10 g L^–1^ yeast extract, 20 g L^–1^ tryptone, 20 g L^–1^ glucose) and *SD* medium (1.7 g L^–1^ yeast nitrogen base, Sangon, China, 5 g L^–1^ ammonium sulfate, Sangon, China, CSM, MP Biomedicals, Solon, OH, USA, 20 g L^–1^ glucose) were used for the activation and culture of the NAN-27 and EMV-8 strain and the host strain BY4741. Sc-URA medium using CSM-URA (uracil single dropout of complete supplement mixture) instead of the CSM of SD medium was used for the activation and batch fermentation of *S. cerevisiae* strains carrying plasmids. Vanillin was added to the medium as indicated. All of the cultures were incubated at 30^°^C.

### Fermentation

A single colony was cultured in 3 ml of SD or SC-URA for 24 h. Next, the cultures were shifted into 20 ml of fresh medium with an OD_600_ of 0.2 overnight culture. Then, the overnight suspension cultures were inoculated into 100 ml flasks with 40 ml of fermentation medium at an initial OD_600_ of 0.2. Then, the batch fermentation was cultured at 30^°^C and 200 rpm.

### Analysis of extracellular vanillin

The concentrations of extracellular vanillin and vanillyl alcohol were measured by an HPLC Waters system e2695 (Waters, USA) prepared with an Xbridge™-C18 column (Waters, USA). The peaks were detected at room temperature by an ultraviolet detector (PDA-2998) at 210 nm with a mobile phase of 40% absolute methanol (Chromatographic grade, Fisher Chemical, USA) supplied at a flow rate of 0.6 ml min^–1^.

### Transcriptome analysis

The precultured NAN-27 and EMV-8 cells in the SD medium were transferred into fresh SD or SD medium containing 8 mM vanillin with an initial OD_600_ of 0.2. The cells were harvested during the log phase (OD_600_ = 1.0) and quenched using liquid nitrogen. The UNIQ-10 TRIzol RNA Purification Kit (Sangon Biotech, China) was used to extract total RNA, from which mRNA was isolated, fragmented, and used as a template to synthesize cDNA. The short fragments were connected with adapters to obtain suitable fragments for PCR amplification. Ultimately, the libraries were sequenced by the Illumina HiSeq™ 4000 (ANNOROAD Genome Beijing, China). The differentially expressed genes were screened out according to the following criteria: fold change ≥ 2 and FDR ≤ 0.001. All of the analyses were performed in biological triplicates. The transcriptome data were deposited in the NCBI Sequence Read Archive (SRA accession number: PRJNA856062).

### Hi-C analysis

EMV-8 and NAN-27 were cultured in *SD* with 8 mM vanillin or without vanillin. When the OD_600_ reached 1.0, the cells were collected for Hi-C. Hi-C libraries were constructed according to previous studies (Belton and Dekker, 2015). The collected cells were cross-linked for 20 min with 3% formaldehyde at room temperature and quenched with 0.375 M glycine for 5 min. The cross-linked cells were homogenized by grinding them to a fine powder in liquid nitrogen to lyse the cell walls. Endogenous nuclease was inactivated with 0.1% SDS, and then chromatin DNA was digested by 100 U *Mbo*I (NEB), marked with biotin-14-dCTP (Invitrogen), and ligated by 50 U T4 DNA ligase (NEB). After reversing the cross-links, the ligated DNA was extracted using a QIAamp DNA Mini Kit (Qiagen) following the manufacturers’ instructions. Purified DNA was sheared into 300–500-bp fragments and further blunt-end repaired, A-tailed, and adaptor-added, followed by purification through biotin–streptavidin-mediated pull-down and PCR amplification. Finally, the Hi-C libraries were quantified and sequenced on the Illumina NovaSeq platform (San Diego, CA, USA) or the MGI-seq platform (BGI, China). The Hi-C data were deposited in the NCBI Sequence Read Archive (SRA accession number: PRJNA855494).

## Results

### Vanillin disturbance or genetic disturbance does not affect the interaction pattern between chromosomes when strains are grown to an OD_600_ of 1.0

**FIGURE 1:**
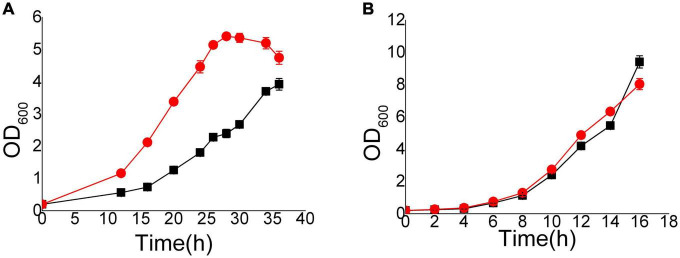
Growth curves of EMV-8 and NAN-27 in medium with 8 mM vanillin stress. The cells were cultured in 40 ml *SD* and added 8 mM vanillin **(A)** or *SD* **(B)** media at 30^°^C, 200 rpm, starting with an initial OD600 of 0.2. Data are the mean values of triplicate tests. Symbols, red circle indicated EMV-8 and black square indicated NAN-27.

We focused on the mechanism of detoxification other than the stress response. Thus, the Hi-C and transcriptome samples were cultured with 8 mM vanillin at the initiation of culture. We chose to collect samples when the OD_600_ reached 1.0 and the concentrations of vanillin were approximately 7.2 mM (EMV-8) to 7.8 mM (NAN-27). The differences between EMV-8 and NAN-27 were obvious at that OD_600_. As the different resistant strains took different times to reach the same phase, EMV-8 and NAN-27 were collected after 12 and 20 h of culture, respectively (Figure 1).

**FIGURE 2:**
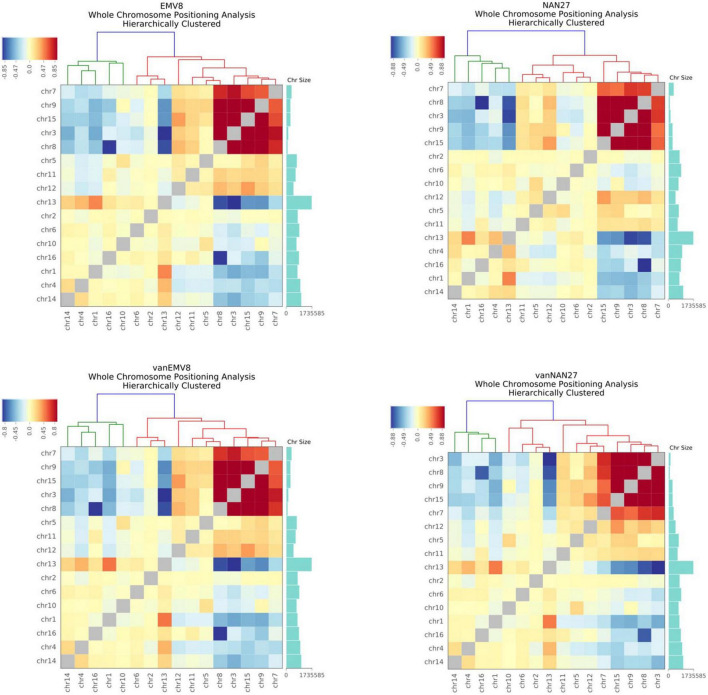
The heat map of the observed/expected number of contacts between all pairs of whole chromosomes in EMV-8 and NAN-27 with or without vanillin stress. Chromosome sizes were annotated, and hierarchical clustering was performed on the X-axis and Y-axis dimensions according to the interaction values between chromosomes. The long chromosomes tend to interact with the long chromosomes, and the short chromosomes tend to interact with the short chromosomes.

Each chromosome occupies a separate area (chromosome territories) in the nucleus, forming a chromatin domain. However, due to the small nuclear space, there will still be contacts between adjacent chromosomes. In human and mouse cells, longer chromosomes tend to be closer to long chromosomes, while chromosomes with smaller lengths tend to contact spatially with small chromosomes (Zhang et al., 2012). We standardize the interaction between the chromosomes to obtain the standardized interaction matrix. The higher the interaction value between two chromosomes, the closer the two chromosomes are in space. A similar interaction pattern exists in both EMV-8 and NAN-27 with or without vanillin, even though there were a total of 450 CDSs with non-synonymous SNPs and 44 CDSs with InDels in strain EMV-8 compared to its parent strain NAN-27 (Wang et al., 2017). The *S. cerevisiae* genome is approximately 12.2 Mb, with 6,275 genes compactly organized on 16 chromosomes. The genome of NAN-27 is approximately 12.02 Mb, with 5,700 genes according to the *de novo* sequencing of NAN-27 (Wang et al., 2017). There were frequent contacts between Chr3 (155,425 bp), Chr7 (370,497 bp), Chr8 (106,760 bp), Chr9 (297,348 bp), and Chr15 (301,727 bp) (Figure 2). These five chromosomes were obviously much shorter than the other chromosomes. Chr13, the longest chromosome in *S. cerevisiae* (1,735,585 bp), has more frequent interactions with Chr1 (771,560 bp), Chr4 (957,666 bp), and Chr14 (1,025,210 bp). In the whole genome, in the log phase, the interaction pattern between chromosomes disturbed by vanillin or genetic mutations was similar to that in mouse cells, in which the longest chromosomes interact with each other more frequently than with the shortest chromosomes and vice versa.

### Topologically associating domain analysis

The nuclear organization of TADs is vital for transcription. Chromatin contacts preferentially occur between loci inside the same TAD rather than between TADs. The regions separating one TAD from another are referred to as boundaries (Eser et al., 2017). Gene expression on the border of a TAD is more active, while gene expression inside a TAD is less active. The TAD boundary is the region enriched with highly expressed genes. There are two types of TAD boundary changes: (a) split refers to a TAD split into two TADs, and a new boundary appears; (b) merge refers to two adjacent TADs that merge into one TAD, and the boundary between the two adjacent TADs disappears. We performed a comparative analysis of TADs between different samples at 2-kb resolution. The TAD boundary changes and relevant gene differential expression are listed in Table 1. We found that 85 TAD borders disappeared in the parent strain NAN-27 after vanillin stress, and 31 TAD borders were newly formed. However, the vanillin-tolerant strain EMV-8 had only 51 TAD borders that disappeared, and 11 TAD boundaries were newly formed after vanillin stress. This finding indicates that in response to vanillin stress, the TAD structure of the vanillin-tolerant strain EMV-8 was more stable than that of the control strain NAN-27. When NAN-27 responded to vanillin stress, the TAD boundaries of 740 genes split or merged, of which 68 genes were differentially expressed. When EMV-8 was stressed by vanillin, the TAD boundaries of 619 genes split or merged, and only 11 genes were differentially expressed. In the comparable group between EMV-8 and NAN-27, only 20 of 670 genes whose TAD boundaries changed exhibited differential expression. However, the style of TAD boundary shifting exhibited no connections with gene expression changes (Table 2). This phenomenon was also observed in other samples (data not shown). In the comparison between EMV-8 and its parent strain NAN-27 under 8 mM vanillin stress, 44 of 830 genes whose TAD boundaries changed exhibited differential expression. The shifting TAD boundaries do not necessarily lead to differential gene expression.

**TABLE 1:** The number of TAD boundaries changing and of the differentially expressed genes caused by TAD border changing.

	NAN-27 response to vanillin	EMV-8 response to vanillin	EMV-8 vs. NAN-27	EMV-8 vs. NAN-27 under 8 mM vanillin
TAD boundaries reforming	32	11	5	2
TAD boundaries disappearing	85	51	32	42
Genes of TAD border changed	740	619	670	840
Different expressed genes caused by TAD border changing	68	10	20	44

**TABLE 2:** The relationship between TAD boundaries shifting and transcription in comparison of EMV-8 and NAN-27.

Gene name	Style	logFC	Function
*IRC18*	Merge	4.72	Protein involved in outer spore wall assembly
*SPO20*	Split	1.86	Required for pro-spore membrane formation during sporulation
*BUB1*	Split	–1.32	Protein kinase involved in the cell cycle checkpoint into anaphase
*DTR1*	Split	1.43	Putative dityrosine transporter of the major facilitator superfamily
*IDP3*	Split	1.87	Peroxisomal NADP-dependent isocitrate dehydrogenase
*KAR2*	Merge	–1.10	ATPase involved in protein import into the ER
*YPK2*	Merge	1.90	Protein kinase participates in the cell wall integrity signaling pathway
*SED1*	Merge	1.17	Stress-induced structural GPI-cell wall glycoprotein
*ACA1*	Split	1.07	Basic transcription factor for carbon source utilization
*UBX6*	Merge	1.22	Ubiquitin regulatory X domain-containing protein
*YPC1*	Split	1.84	Alkaline ceramidase
*CCW14*	Merge	1.28	Covalently linked cell wall glycoprotein
*MEI4*	Split	3.88	Meiosis-specific protein involved in forming double-strand break formation
*AFR1*	Merge	1.35	Required for pheromone-induced projection formation
*YMR103C*	Merge	1.56	Uncharacterized
*CRH1*	Split	1.03	Chitin trans-glycosylase
*POL2*	Merge	–1.07	Catalytic subunit of DNA polymerase (II) epsilon
*ECM19*	Merge	1.06	Protein of unknown function
*SMP1*	Split	1.17	Transcription factor related to osmotic stress response
*THI73*	Split	1.16	Putative plasma membrane permease

Style: TAD boundaries shifting style logFC referred in comparison of EMV-8/NAN-27 which is less than 0 means downregulation and large than 0 means upregulation at the transcription level; *P*-value ≤ 0.001.

### Analysis of motifs enriched at topologically associating domain boundaries

The borders of TADs are rich in transcription factors related to promoters, transcription initiation sites, housekeeping genes, and tRNA genes, which are important for maintaining the stability of TAD structure and stability (Dixon et al., 2012). Thus, the enrichment analysis of the TAD boundary motifs was performed in four samples of EMV-8 and NAN-27 with or without vanillin stress (Table 3). The outcomes indicated that all the samples had TAD enrichment motifs of *SFL1*, *STB3*, and *NHP6A/B*. Sfl1p is a transcriptional repressor of flocculation-related genes, and its deletion enhances pseudohyphal and invasive growth (Fujita et al., 1989; Robertson and Fink, 1998). Stb3p is a ribosomal RNA processing element (RRPE)-binding protein and participates in the glucose-induced transition from quiescence to growth (Kasten and Stillman, 1997; Liko et al., 2007, 2010). *NHP6A* and its paralog *NHP6B* were reported to bind to and remodel nucleosomes, recruit facilitates chromatin transcription (FACT) and other chromatin remodeling complexes to chromosomes, and ensure transcriptional initiation fidelity of some tRNA genes (Ruone et al., 2003; Kassavetis and Steiner, 2006; Stillman, 2010). The above four proteins bound to these common motifs may play vital roles in TAD formation in *S. cerevisiae*. NANA-27 has its own specific motif, *AZF1*, with or without vanillin stress. Azf1p is a zinc-finger transcription factor that activates the transcription of genes involved in carbon metabolism and energy production from glucose (Newcomb et al., 2002). EMV-8 with no stress enriches the *SUM1* motif at its TAD boundary, which encodes a transcriptional repressor that regulates middle-sporulation genes and is involved in telomere maintenance

**TABLE 3:** The top five motifs enriched at TAD boundaries in EMV-8 and NAN-27 with or without vanillin.

Motif	EMV-8	EMV-8 under vanillin stress	NAN-27	NAN-27 under vanillin stress
*SFL1*	281/303	267/297	276/309	285/309
*STB3*	278/303	274/297	284/309	285/309
*NHP6A*	269/303	266/297	274/309	276/309
*NHP6B*	268/303	271/297	276/309	278/309
*SUM1*	268/303	NA	NA	NA
*SFP1*	NA	263/297	NA	NA
*AZF1*	NA	NA	281/309	279/309

**FIGURE 3:**
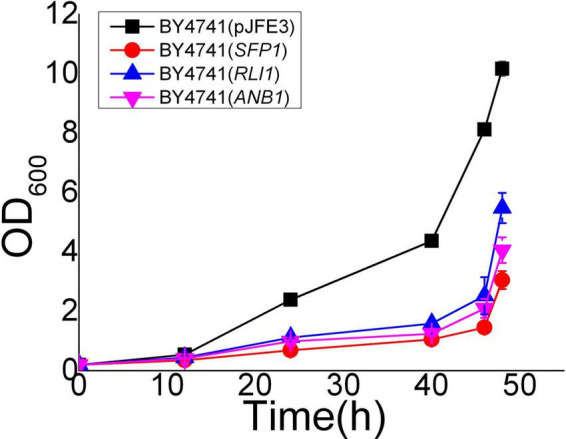
Vanillin resistance profiles of recombinant strains which overexpress translation related genes in laboratory strain BY4741 grown in SC-URA liquid medium supplemented with 6 mM vanillin. Cultivations were performed at 30^°^C. The error bars indicated the standard deviation of independent triplicates.

Number 1/number 2 indicated border number containing motif/total border number. NA, indicated not found.

(Lempiäinen et al., 2000; Edmonds et al., 2004). At the TAD borders of EMV-8 under vanillin stress, there are a large number of *SFP1* binding sites. Sfp1p can regulate the transcription of ribosomal proteins and ribosomal synthesis genes, as well as regulate the response to nutrients and stress (Lempiäinen et al., 2000; Marion et al., 2004). Although the expression level of *SFP1* did not change significantly in EMV-8, under vanillin stress, some translation elongation factors in EMV-8, such as *ANB1* and ribosome synthesis factor *RLI1*, were significantly upregulated. This may have a positive effect on the improvement of EMV-8 vanillin resistance because our previous proteomic analysis showed that ribosomal proteins and rRNA processing-related proteins in *S. cerevisiae* BY4741 were significantly decreased in response to vanillin stress (Cao et al., 2021). However, the overexpression of *ANB1*, *RLI1*, and *SFL1* in the laboratory strain BY4741 did not improve *S. cerevisiae* (Figure 3).

### Differences in genes interacting with *YRR1* caused by the InDel mutation of *YRR1*

**FIGURE 4:**
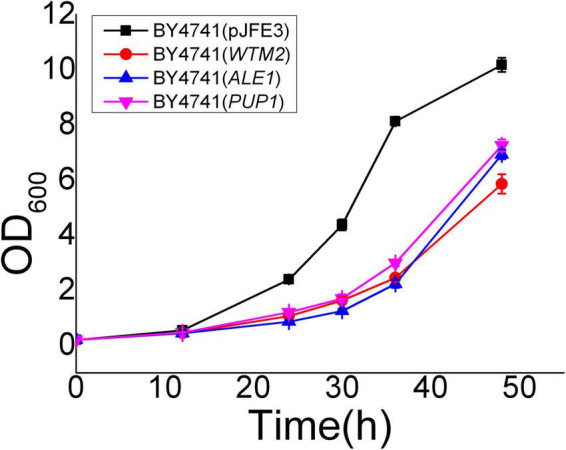
Vanillin resistance profiles of recombinant strains grown in SC-URA liquid medium supplemented with 6 mM vanillin. Cultivations were performed at 30^°^C. All the data were mean value ± standard deviation of independent triplicates.

Yrr1 has an InDel mutation in the vanillin-resistant strain EMV-8; the 409th adenylate deoxyribonucleotide of its ORF is deleted, and a frameshift mutation occurs, which causes the translation to stop prematurely at the 141st amino acid. Compared with NAN-27, *YRR1* in EMV-8 had 14 specific interaction sites (Table 4). Two of the 14 sites also interacted with *YRR1* when EMV-8 was under vanillin stress. These two sites were EMV-8 specific, probably caused by the mutation of *YRR1*. This mutation causes *YRR1* to produce new interaction sites for three genes, namely, *WTM2*, *PUP1*, and *ALE1*. *WTM2* encodes a transcriptional regulator that regulates meiosis and the expression of nucleotide reductase, which can respond to DNA replication pressure (Pemberton and Blobel, 1997; Tringe et al., 2006), *PUP1* encodes the 20S proteasome subunit (Haffter and Fox, 1991), and *ALE1* encodes lysophospholipid acylase (Riekhof et al., 2007). However, these three genes did not show differential expression at the transcriptional level, and the biological significance of this is not clear. The overexpression of three genes in the laboratory strain BY4741 did not improve the vanillin resistance of *S. cerevisiae* (Figure 4).

**TABLE 4:** The specific interaction sites of *YRR1* in EMV-8, compared to NAN-27.

Chromosome number	Site
14	421,500
14	299,500
14	435,500
14	309,500
12	249,500
14	398,500
14	418,500
10	406,500
14	405,500
14	405,500
6	142,500
14	352,500
4	825,500
14	429,500

## Discussion

A high vanillin stress-tolerant strain, EMV-8, was derived from NAN-27 by EMS mutation and adaptive evolution in lignocellulosic hydrolysates. Even though EMV-8 has a total of 450 CDSs with non-synonymous SNPs and 44 CDSs with InDels compared to its parent strain NAN-27, the chromosome interaction patterns were similar in both strains when they were collected in the log phase with an OD_600_ of 1.0. Similarly, the interaction of chromosomes disturbed by vanillin stress in the log phases of EMV-8 and NAN-27 exhibited the same pattern: short chromosomes interact more frequently with short chromosomes, and long chromosomes interact more frequently with long chromosomes. After the lag phase of adaptation, the differences in chromosome interactions in the log phase were probably not as obvious as the initiation of vanillin addition. The interactions of chromosomes perhaps exhibited significant differences in the lag phase. However, these differences were likely due to stress responses other than detoxification or adaptation.

A similar interaction pattern also appeared in mouse cells (Zhang et al., 2012). Shao et al. (2018) found that the local chromatin interactions of BY4742 and the synthetic single-chromosome yeast strain SY14, at least at the level of gene loci, were very similar. It was presumed that the interaction patterns of chromosomes were not affected by the structures of the chromosomes and that an arithmetic model of calculating interactions by Hi-C was perhaps not suitable.

In response to vanillin stress, the TAD boundary changes in EMV-8 were less than those in its parent strain NAN-27. The TAD structure of EMV-8 was more stable after long-term evolution in lignocellulosic hydrolysates than NAN-27. The differences in TAD boundaries between the two strains did not result in the corresponding theoretical differential expression. In Shao et al. (2018) research, a single-chromosome yeast SY14 was artificially synthesized by chromosomal fusions involving 16 chromosomes, and its overall chromosomal 3D structure was changed markedly compared to its parent strain BY4742. However, the transcriptome of the single-chromosome cells was nearly identical to that of the parental cells (Shao et al., 2018). This outcome was inconsistent with studies by Spilianakis et al. (2005) and Meaburn and Misteli (2007) who found that the localization of a chromosome in the nucleus and interchromosome interactions affect gene expression. Our outcome also confirmed that transcriptomic expression showed a negligible relationship with chromosomal 3D structure or TAD boundaries shifting in the log phase. The samples should be further checked at other time points of growth, such as the lag phase, or checked with higher concentrations of vanillin stress. The other possibility is that the precision of Hi-C is not sufficient for *S. cerevisiae*, whose genome is only 12.2 Mb.

There were four motifs enriched at the TAD boundaries in four samples (EMV-8 and NAN-27 with or without vanillin): *SFL1*, *STB3*, and *NHP6A/B*. These genes were probably related to the formation of TAD boundaries. Their functions in TAD boundary formation need to be further studied. In response to the inactivation of the TOR pathway, cell stress, or nutrient limitation, Sfp1p relocalizes to the cytoplasm and inhibits ribosomal protein (RP) gene expression. Sfp1p plays a crucial role in modulating cell growth and RP gene expression in response to environmental cues (Marion et al., 2004). The resistance to vanillin of the *SFP1*-deleted BY4741 strain decreased significantly (data not shown). The motif of *SFP1* was enriched only in TAD boundaries of EMV-8 with vanillin stress. This result indicated that EMV-8 could probably regulate the expression of RP genes promptly to adapt to vanillin stress, as Cao et al. (2021) found that *S. cerevisiae* adapted to vanillin stress by repressing ribosomal protein abundance to save energy.

*YRR1* in EMV-8 had an InDel mutation and lost its function, and its absence was confirmed to benefit vanillin tolerance. The InDel mutation caused two new interaction sites for three genes, *WTM2*, *PUP1*, and *ALE1*. However, the expression levels of the three genes exhibited no differences. There were no obvious regulatory relationships between mutated *YRR1* and the three genes. Du et al. (2017) found that some long-distance chromosomal interactions can affect gene expression in yeast by inserting the *MET3* promoter into genomic loci and building an assay to screen for functional long-distance interactions that affect the average expression level of a reporter gene. Perhaps more attention should be paid to promoters rather than functional genes. This assay could be used in EMV-8 and NAN-27 to screen the differences in long-distance chromosomal interactions.

## Conclusion

We performed a conjoint analysis of Hi-C and compared transcriptomes in the vanillin-resistant strain EMV-8 and its parent strain NAN-27 with or without vanillin. In the log phases of EMV-8 and NAN-27, the chromosome contact patterns disturbed by vanillin or genetic mutations were similar to those in mouse cells. Short chromosomes tend to contact short chromosomes, and long chromosomes tend to contact long chromosomes. In response to vanillin stress, the TAD structure of the vanillin-tolerant strain EMV-8 was more stable than that of its parent strain NAN-27. TAD boundary changes have a negligible effect on gene transcription in yeast. The overexpression of genes whose motifs were enriched in the TAD boundaries of EMV-8 and NAN-27 did not affect vanillin resistance in yeast. The InDel mutation of *YRR1* in EMV-8 resulted in the formation of two new interaction sites for three related genes. The overexpression of these three genes did not affect the vanillin resistance of yeast. This research explored the effects of genetic mutations and vanillin disturbance on three-dimensional chromosome organization and dynamic changes in TADs in the log phase of growth.

## Data availability statement

The datasets presented in this study can be found in online repositories. The names of the repository/repositories and accession number(s) can be found in the article/supplementary material.

## Author contributions

XW and XB conceived and designed the study. XW and BY participated in the design of experiments and data collection, analyzed the data, and drafted the manuscript. XW performed genome sequencing and RNA-seq data analysis. BY conducted the fermentation and the construction of recombinant strain. WZ and WC conducted the samples for Hi-C and transcriptome. YS, ZL, and XB supervised and coordinated the overall study. All authors read and approved the final manuscript.
